# Interrelationships Between Plasma Levels of Brain Natriuretic Peptide and Prolonged Symptoms Due to Long COVID

**DOI:** 10.3390/jcm14030817

**Published:** 2025-01-26

**Authors:** Yohei Masuda, Yuki Otsuka, Kazuki Tokumasu, Hiroyuki Honda, Yasue Sakurada, Yui Matsuda, Yasuhiro Nakano, Ryosuke Takase, Daisuke Omura, Toru Hasegawa, Keigo Ueda, Fumio Otsuka

**Affiliations:** Department of General Medicine, Okayama University Graduate School of Medicine, Dentistry and Pharmaceutical Sciences, 2-5-1 Shikata-cho, Kita-ku, Okayama 700-8558, Japan; yoheimasuda@s.okayama-u.ac.jp (Y.M.); otsuka@s.okayama-u.ac.jp (Y.O.); tokumasu@okayama-u.ac.jp (K.T.); ppgf1hrd@s.okayama-u.ac.jp (H.H.); pzaf6h9w@s.okayama-u.ac.jp (Y.S.); phvw0350@okayama-u.ac.jp (Y.M.); y-nakano@okayama-u.ac.jp (Y.N.); p4v05asb@okayama-u.ac.jp (R.T.); me20011@s.okayama-u.ac.jp (D.O.); pwcp0od9@s.okayama-u.ac.jp (T.H.); kuedampo@okayama-u.ac.jp (K.U.)

**Keywords:** brain fog, brain natriuretic peptide (BNP), COVID-19, fatigue, long COVID, memory impairment, post-COVID-19 conditions

## Abstract

**Objectives:** Evidence for the usefulness of biomarkers that aid in diagnosis, assessment of severity, and prediction of prognosis in patients with long COVID is limited. The aim of this study was to clarify the characteristics of brain natriuretic peptide (BNP) in long COVID. **Methods:** We conducted a retrospective observational study of patients who visited the COVID-19 aftercare outpatient clinic at Okayama University Hospital from February 2021 to April 2024. **Results:** A total of 428 patients were enrolled in this study, and the patients were divided into a group with normal BNP (n = 314, ≤18.4 pg/mL) and a group with increased BNP (n = 114, >18.4 pg/mL). The long COVID group with increased BNP had a higher proportion of females (44.3% vs. 73.7%, *p* < 0.01) and an older median age (38 vs. 51 years, *p* < 0.01). Fatigue and brain fog were commonly manifested in both groups, while dyspnea was a more frequent complaint in the group with increased BNP. Various symptoms including fatigue, palpitations, and taste and/or olfactory disorders were associated with elevated BNP (23 to 24 pg/mL). Memory impairment was also linked to higher BNP (OR: 2.36, *p* = 0.05). In long COVID patients, plasma BNP elevation appears to be more pronounced in females and is often related to cardiogenic factors, in which inflammatory responses are also involved. **Conclusions:** Plasma BNP measurement may be useful for evaluating the severity of long COVID, especially in female patients and those with respiratory symptoms and/or memory impairment.

## 1. Introduction

Long COVID, a disabling condition that presents various symptoms after COVID-19, is estimated to affect nearly 60 million people worldwide [1]. Long COVID leads to a decline in the quality of life of patients [2] and often results in difficulty in working [3]. In February 2021, we established a COVID-19 aftercare clinic (CAC) for patients suffering from long COVID, and we have been operating the clinic for the past three years [4].

Several clinical indicators for inflammatory and endocrine dysfunctions in patients with long COVID have been established [5,6,7,8]. However, a specific biomarker for prognosis of long COVID has not been established [9], though such a biomarker is needed for assessing treatment response and estimating prognosis [10]. Long COVID affects multiple organs and systems [11], making it difficult to explain its pathophysiology by a single mechanism, and there are many subtypes [10]. We consider that multiple biomarkers are needed to explain each pathophysiology of long COVID [12]. We previously revealed some relationships between symptoms and biomarkers including a relationship between myalgic encephalomyelitis/chronic fatigue syndrome (ME/CFS) and ferritin [13] and a relationship between brain fog and compliments shown as CH50 [14].

SARS-CoV-2 infection induces systemic effects including cardiovascular involvement and related complications. Cardiac myocytes secrete brain natriuretic peptide (BNP) in response to cardiac wall stress [15]. BNP is clinically useful for diagnosing and estimating the prognosis of heart failure [16,17]. It has been suggested that cardiac injuries induced by SARS-CoV-2 are involved in the “fatigue” related to COVID-19 and/or long COVID. In the acute phase of COVID-19, it has been shown that BNP level is a possible predictor for prognosis [18] and that BNP level is elevated in patients with severe COVID-19 [19], whereas another study showed that the viral infection itself has no direct impact on cardiac markers such as BNP and troponin [20].

On the other hand, a study on long COVID showed that N-terminal pro-B-type natriuretic peptide (NT-proBNP), a biomarker that is commonly associated with heart failure and is produced in response to cardiac muscle cell stretching, was not related to impaired cardiac function but was related to hand-grip strength in long COVID patients with ME/CFS [21]. Thus, there has been no direct evidence of the utility of measuring plasma BNP for long COVID cases. In the present study, we focused on plasma BNP concentrations in patients with long COVID and attempted to validate the clinical utility of BNP as a biomarker for long COVID.

## 2. Patients and Methods

### 2.1. Study Design and Clinical Evaluation of Long COVID

This study was a retrospective observational study conducted at Okayama University Hospital, a university hospital in Japan. We established a CAC at this hospital on 15 February 2021 and have been providing medical care for long COVID patients for three years [4]. In this study, long COVID was characterized by symptoms that persisted for more than four weeks following the onset of COVID-19, as we previously reported [22]. The severity of the acute phase of COVID-19 was assessed on the basis of the criteria outlined by the Ministry of Health, Labour and Welfare in Japan [23]. Physicians conducted detailed interviews about symptoms during face-to-face interviews with patients.

### 2.2. Inclusion of Patients

**Figure 1:**
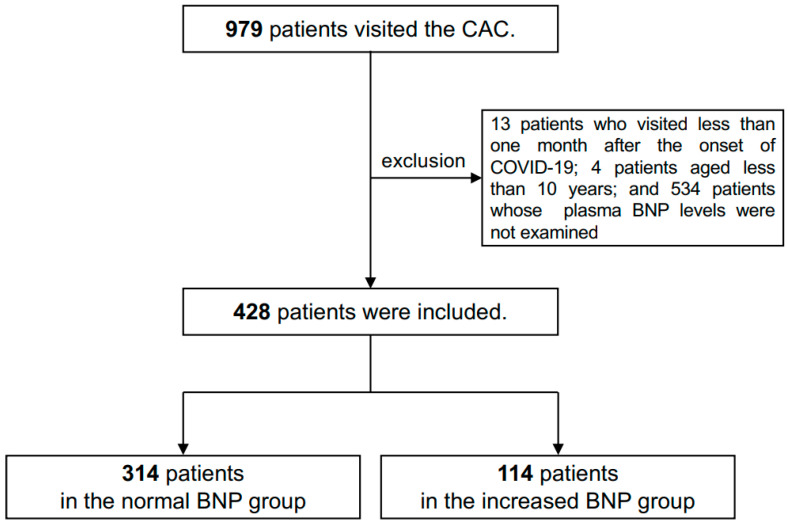
Flowchart of patient screening and inclusion. Of the 979 patients who visited the CAC, 428 were included in the analysis. Among them, 314 were assigned to the normal BNP group and 114 were assigned to the increased BNP group.

In this study, we reviewed the medical records of all patients who visited the CAC from February 2021 to April 2024. We defined long COVID as “persistence of several symptoms for more than one month after the onset of COVID-19” [4,24]. A diagnostic test confirming SARS-CoV-2 infection was not required as evidence of infection [1]. We included patients aged 10 years or older due to ethical considerations such as the difficulty in obtaining informed consent from patients younger than 10 years of age. Among the 979 patients who visited the CAC during that period, 13 patients who presented within one month of COVID-19 onset and 4 patients under the age of 10 years were excluded from the analysis. After excluding 534 patients who did not have their plasma BNP levels examined, the remaining 428 patients with long COVID were included in the present study (Figure 1).

### 2.3. Evaluated Factors

Patients were divided into two groups based on plasma BNP levels: a normal BNP group with BNP levels ≤ 18.4 pg/mL (314 patients) and an increased BNP group with BNP levels > 18.4 pg/mL (114 patients) [25]. Information on gender, age, body mass index (BMI), systolic blood pressure, diastolic blood pressure, pulse rate, smoking and alcohol drinking habits, days from COVID-19 onset to the first visit, hospital admission, oxygen therapy, steroid therapy, severity of the acute phase of COVID-19, COVID-19 vaccination history, and clinical symptoms of long COVID was obtained from medical records.

### 2.4. Measurements of BNP and Laboratory Markers

Blood sampling was performed in a sitting position when the patient visited our CAC. BNP levels were measured when clinically necessary, at the discretion of each physician. Routine blood samples were analyzed using the auto-analyzer system in the central laboratory of our facility. Plasma BNP was measured by a chemiluminescent enzyme immunoassay (CLEIA) using the LUMIPULSE L2400 and LUMIPULSE Presto BNP kit (FUJIREBIO Inc., Tokyo, Japan). In addition to BNP, other laboratory data including data for hemoglobin (Hb), inflammatory markers (C-reactive protein (CRP) and erythrocyte sedimentation rate (ESR)), liver functions (albumin (Alb), aspartate aminotransferase (AST), alanine aminotransferase (ALT), and alkaline phosphatase (ALP)), and renal functions (estimated glomerular filtration rate (eGFR)) were obtained from medical records and analyzed in this study.

### 2.5. Statistical Analysis

All analyses were conducted using Stata Statistical Software: Release 18 (StataCorp LLC, College Station, TX, USA). The Mann–Whitney U test was used for comparing continuous variables. Fisher’s exact test and Pearson’s chi-square test were used for categorical variables. Logistic regression analysis was used for multivariate analysis and calculation of odd ratios (ORs) and 95% confidence intervals (CIs). *p*-values of * *p* < 0.05 and ** *p* < 0.01 were considered statistically significant.

### 2.6. Ethical Approval

The study protocol received approval from the Okayama University Hospital Ethics Committee (No. 2105-030) and adhered to the principles of the Declaration of Helsinki. Study information was made available on our hospital’s website, allowing patients the opportunity to opt out if desired. Informed consent was not required from the patients as all data used in this study were anonymized.

## 3. Results

Of the 428 patients, 114 patients (26.6%) were included in the increased BNP group and 314 patients (73.4%) were included in the normal BNP group. The characteristics of patients in each group are shown in Table 1. There were 139 female patients (44.3%) in the normal BNP group and 84 female patients (73.7%) in the increased BNP group, indicating a significantly larger proportion of female patients in the increased BNP group (*p* < 0.01). The median ages of patients in the normal BNP group and increased BNP group were 38 and 51 years, respectively. Patients in the increased BNP group were significantly older (*p* < 0.01).

Although median systolic blood pressure was significantly higher in the increased BNP group (128 mmHg) than in the normal BNP group (121 mmHg), there was no significant difference in diastolic blood pressure or pulse rate between the two groups. Median BMI was approximately 22 kg/m^2^ in both groups, with no significant difference. As for the patients’ lifestyles, the increased BNP group had a larger percentage of patients with a smoking habit (28.9% vs. 23.6%) and a higher proportion of patients with a habit of alcohol consumption (26.3% vs. 19.4%) than the percentages of such patients in the normal BNP group, but the differences were not statistically significant (Table 1).

As for the care for the acute phase of COVID-19, the percentage of patients in the increased BNP group who were hospitalized was significantly higher than that in the normal BNP group (9.65% vs. 4.14%, *p* < 0.05). Also, the increased BNP group included much higher percentages of patients who received oxygen therapy and steroid therapy and included a higher percentage of patients with severe COVID-19 than those in the normal BNP group, but the differences were not statistically significant. Vaccination status was not significantly different between the two groups. The periods from the onset of COVID-19 to the initial visit to the CAC were longer in the increased BNP group than in the normal BNP group (Table 1).

**Figure 2:**
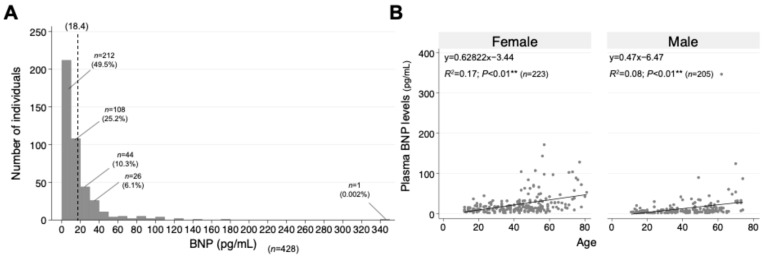
Distribution of plasma BNP levels in long COVID patients. (**A**) Distribution of total BNP levels in the study population. The histogram displays a right-skewed distribution of all 428 enrolled patients. (**B**) Age- and gender-related distributions of BNP levels. Scatter plot with a correlation line is stratified by gender, including 233 female and 205 male patients. Differences were considered statistically significant at ** *p* < 0.01.

Characteristics of the BNP levels in all of the patients are shown in Figure 2. The histogram of BNP levels in the study population showed a right-skewed distribution, with approximately 70% of the patients having BNP levels below 20 pg/mL. Only one patient had an extremely high level of BNP exceeding 340 pg/mL, while all other patients had BNP levels below 180 pg/mL (Figure 2A). According to the correlation with age by gender, BNP levels increased with age in both males and females, and this trend was more pronounced in females (Figure 2B).

**Figure 3:**
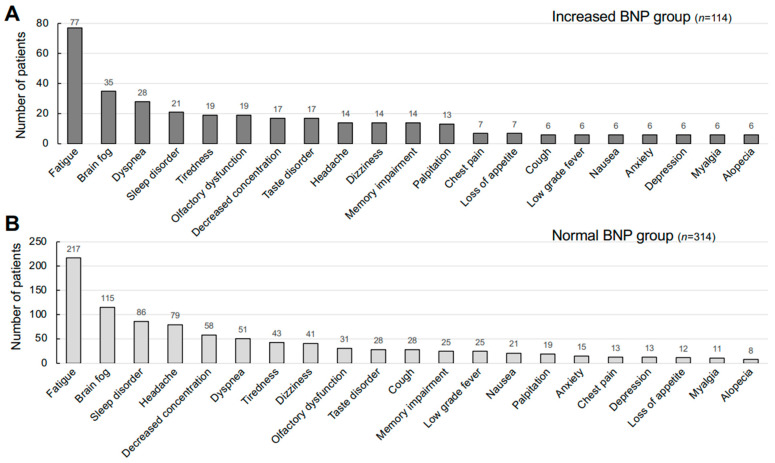
Clinical symptoms complained by the long COVID patients with or without increased plasma BNP levels. Frequent symptoms in 114 patients in the increased BNP group (**A**) and those in 314 patients in the normal BNP group (**B**) were graphed.

**Figure 4:**
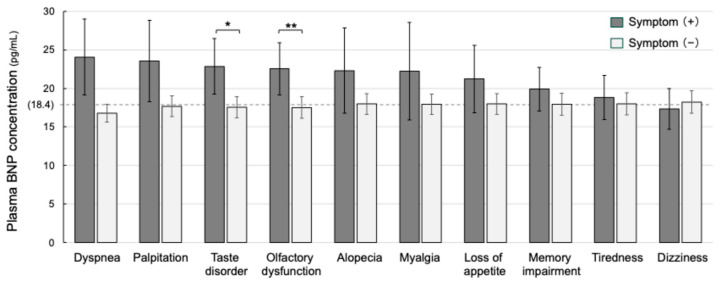
Plasma BNP concentrations in the long COVID patients with or without major individual symptoms. Mean plasma concentrations of BNP and standard error of the mean (SEM) were graphed according to the presence or absence of typical symptoms related to long COVID. The Mann–Whitney U test was performed, and the differences were considered statistically significant at * *p* < 0.05 and ** *p* < 0.01.

The number of individuals complaining of each symptom in the two groups is shown in Figure 3. Fatigue and brain fog were the most common symptoms in both groups, while dyspnea was the third most frequent symptom only in the increased BNP group. Olfactory dysfunction and taste disorders were also common symptoms in the increased BNP group. As shown in Figure 4, a comparison of the average BNP levels by the presence of these symptoms in all of the patients showed that patients with taste disorders and olfactory dysfunction had significantly higher BNP levels than those in patients without these symptoms (22.9 pg/mL vs. 17.6 pg/mL; *p* < 0.05 and 22.5 pg/mL vs. 17.5 pg/mL *p* < 0.01, respectively). Although the patients with dyspnea and patients with palpitations also had much higher average BNP levels (23 to 24 pg/mL) than those in patients without these symptoms, the differences were not statistically significant. Other symptoms that were associated with relatively high average BNP levels were dyspnea (24.1 pg/mL), palpitation (23.6 pg/mL), alopecia (22.3 pg/mL), myalgia (22.2 pg/mL), loss of appetite (22.2 pg/mL), memory impairment (19.9 pg/mL), and tiredness (18.8 pg/mL) (Figure 4).

**Figure 5:**
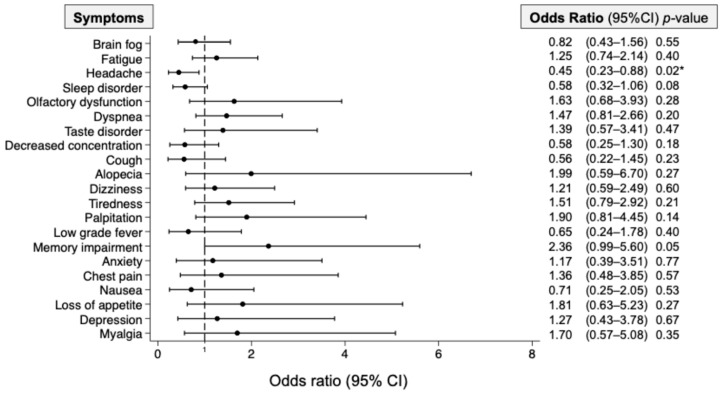
Symptoms related to the increase in plasma BNP levels in the patients with long COVID. A logistic regression analysis was performed, and the odds ratios related to increases in plasma BNP levels were evaluated for each major symptom of long COVID. The differences were considered statistically significant at * *p* < 0.05. CI: confidence interval.

A logistic regression analysis of the 21 major symptoms revealed that there were no symptoms with significantly higher OR for increased BNP levels (Figure 5). However, in point estimates, representative symptoms of long COVID including fatigue (OR: 1.25), olfactory dysfunction (1.63), dyspnea (1.47), taste disorder (1.39), alopecia (1.99), palpitations (1.90), memory impairment (2.36), and chest pain (1.36) tended to have higher ORs. Among these, the symptom of memory impairment had a significantly higher OR (*p* = 0.05) in the present study. Conversely, headache had a significantly lower OR (OR, 0.45; 95% CI, 0.23–0.88; *p* < 0.05). As shown in Table 2, the results of the logistic regression analysis for patients’ background showed that older age (OR, 1.06; 95% CI, 1.04–1.07; *p* < 0.01), and female gender (OR, 3.80; 95% CI, 2.22–6.50; *p* < 0.01) had significantly higher ORs for increased BNP.

We compared other laboratory data between the normal BNP group and the increased BNP group, as shown in Table 3. Statistically significant differences (*p* < 0.01) were observed in the mean levels of hemoglobin (14.7 g/dL vs. 13.5 g/dL), albumin (4.52 g/dL vs. 4.24 g/dL), and ESR (8.57 mm/h vs. 14.93 mm/h). Thus, the inflammatory responses were related to the increases in plasma BNP concentrations.

## 4. Discussion

In the present study, we investigated the relationships between long COVID symptoms and concentrations of plasma BNP. Plasma BNP levels were higher than 18.4 pg/mL in 114 (26.6%) of the patients. In the present study, the plasma BNP concentrations tended to increase with age, particularly in females. Since BNP levels have been shown to be generally elevated in males in a Japanese cohort [26], there might be some other reasons why the plasma BNP levels were elevated especially in elderly female patients in the present study.

Herein, we propose two independent etiologies for the elevation of plasma BNP levels in long COVID patients. The first is a cardiogenic etiology, which is widely known [27]. COVID-19 causes cardiac impairments in both the acute and long COVID phases [11] including myocarditis, acute coronary syndrome, heart failure, thromboembolism, and arrhythmias [28]. Given the recent evidence suggesting that the levels of plasma BNP do not always represent cardiac abnormalities at one year after SARS-CoV-2 infection [29], BNP alone should not be used to assess cardiac outcomes in long COVID patients, though it provides temporary information about cardiac involvement. Another etiology for the elevation of BNP is the existence of inflammation. Elevated levels of inflammatory cytokines, such as interleukin-6 and tumor necrosis factor-α, have been observed in patients with long COVID [10,30]. Additionally, there is a positive association between the levels of these cytokines and plasma BNP even in the absence of cardiovascular diseases [31]. In the context of long COVID, BNP might not be useful as a cardiac-specific biomarker but instead might be useful as a biomarker for predicting inflammation-induced symptoms. Considering that BNP is also produced by ischemic skeletal muscle satellite cells, functioning as a paracrine regulator of vasodilation and/or vascular regeneration [21], some extra-cardiovascular factors might also be involved in the changes in plasma BNP levels in long COVID patients.

Our study revealed that two common symptoms of long COVID, fatigue and brain fog, occur frequently regardless of BNP levels. Patients with dyspnea, palpitations, taste disorders, and olfactory disorders had higher BNP levels than those in patients with other symptoms. Conversely, patients with headaches and sleep disorders tended to have rather low BNP levels, while patients with memory impairment tended to have high BNP levels. In a previous study performed by Mottram et al. [32], left ventricular diastolic dysfunction and increased BNP levels were found to be correlated in patients with hypertension and exertional dyspnea. To unravel the relationship between cardiac function and elevated BNP in patients presenting with dyspnea in our cohort, we should consider a cardiac function workup such as transthoracic echocardiography in a future study.

In the context of palpitations, arrhythmias such as chronic atrial fibrillation and paroxysmal supraventricular tachycardia may cause elevation of BNP levels [33,34]. In a clinical setting, a Holter electrocardiogram and/or cardiopulmonary exercise test to detect underlying arrhythmias would be diagnostic in a certain population of long COVID patients with increased BNP [35]. The results of our study further showed that elevated BNP levels are more common in women. Tudoran et al. demonstrated that long COVID is associated with ventricular diastolic dysfunction, which likely persists for more than six months and is more frequently observed in women [36]. The findings of our study may reflect the same correlation.

A recent study in a large prospective cohort of patients who were hospitalized due to COVID-19 showed that serum NT-proBNP levels were significantly associated with short-term prognosis as well as long-term prognosis [37]. In that study, increased NT-proBNP levels were associated not only with in-hospital mortality but also with poor prognosis of mortality 1 year after the infection. One meta-analysis also suggested that NT-pro BNP is associated with COVID-19 severity and in-hospital death [38]. Thus, the concentration of NT-proBNP, in addition to that of BNP, is a possible predictor for a poor prognosis in survivors of the acute phase of COVID-19, which could be linked to the occurrence of long COVID. Further research is needed to confirm the impact of these cardiac markers on long-term prognosis in survivors of acute-phase COVID-19. Other cardiac biomarkers, such as troponin T and CK-MB, also have the potential to estimate disease severity and mortality in COVID-19 patients. These biomarkers are also being considered for their role in monitoring long COVID [39].

In the context of pediatric COVID-19 patients, multisystem inflammatory syndrome in children (MIS-C) is considered to be a severe condition secondary to COVID-19. Chakraborty et al. showed that elevated levels of both BNP and troponin T at admission were associated with a decrease in left ventricular ejection fraction (LVEF) at admission but not at discharge. Our findings should not be applied to patients with MIS-C or pediatric long COVID patients [40].

We have been investigating the relationships between symptoms of long COVID and potential biomarkers [13,14,22,41]. In a study in which the link between orthostatic intolerance (OI) and long COVID was examined [41], approximately 40% of the patients with suspected OI tested positive during an active standing test. Palpitations were a common symptom in the group with positive active standing tests. The symptoms of OI observed in that study closely resemble those of patients in the increased BNP group in this study. This finding highlights the need for further studies to explore the relationship between OI and BNP.

In a previous study, we showed an association between ME/CFS and long COVID [22], with approximately 8% of the patients meeting the criteria for ME/CFS. Patients with both ME/CFS and long COVID exhibited symptoms similar to those of patients in the increased BNP group such as fatigue and brain fog. Taste and olfactory dysfunctions are also common symptoms in long COVID. Taste receptors and olfactory receptors are considered to be vulnerable to inflammation, and loss of these receptors leads to symptoms [42,43]. Memory impairment is also considered to be induced by neuroinflammation of the hippocampus [44]. This is consistent with BNP elevation in our patients, with these symptoms being secondary to inflammation. Thus, in patients with long COVID, plasma BNP elevation seems likely to be involved in not only cardiogenic factors but also some inflammatory issues. The results of this study suggested that plasma BNP measurement is applicable for evaluating the severity of long COVID, especially in female patients and those with cardio-respiratory symptoms and/or memory impairment, although further investigation in this cohort is also warranted.

It is challenging to provide specific recommendations for evaluating cardiac function based on BNP levels, as cardiac comorbidities were not assessed in this trial. However, given that long COVID is associated with an increased risk of cardiac comorbidities, we recommend adopting a lower threshold for initiating diagnostic workups. These may include transthoracic echocardiography or Holter electrocardiography, guided by symptoms such as dyspnea, palpitations, or chest pain, rather than relying solely on BNP levels. Additionally, BNP is a valuable tool for ruling out heart failure. Commonly used thresholds include BNP levels below 100 pg/mL and NT-proBNP levels below 300 pg/mL [16], which differ from the definition of increased BNP in this trial (greater than 18.4 pg/mL). Slight elevations in BNP should not be overly associated with heart failure, particularly in asymptomatic patients. In the context of heart failure, initiating guideline-directed medical therapy [45] based solely on BNP levels is unnecessary. Instead, standard heart failure management practices should be applied.

In the context of the correlations between BNP and other laboratory data, our results further suggested that certain biomarkers are associated with BNP elevation. It has been shown that decreases in hemoglobin and albumin levels, as well as an increase in ESR, are possible biomarkers for long COVID [30]. These findings align with the results of our study, suggesting that increased BNP is involved in inflammatory responses in long COVID and further suggest that BNP may also serve as a predictive factor for long COVID.

Our study has several strengths. This study is the first study to evaluate the relationships between long COVID symptoms and BNP. We validated our findings in long COVID patients with non-cardiac manifestations, and the results were consistent with our thesis that inflammation leads to elevation of BNP. On the other hand, our study also has some limitations. First, our study was a single-center retrospective cohort study with a small sample size. To establish strong evidence, we should conduct a multicenter prospective study. Second, we did not consider cardiac comorbidities as patient baseline characteristics, and we did not have information on baseline BNP levels. We could not assess the change in BNP concentrations over time. In future studies, we should evaluate arrhythmias and heart failure in patients with increased BNP levels who have dyspnea or palpitations. For patients with inflammation-induced symptoms such as olfactory and taste disorders and memory impairment, we should follow BNP trends and functional outcomes over time. Finally, since we began measuring plasma BNP in September 2022, almost all the patients of our cohort were infected with Omicron variants of SARS-CoV-2 (409 cases, 95.6%), and therefore, we could not compare variant-dependent trends of BNP changes.

In conclusion, our results revealed interrelationships between symptoms and plasma BNP levels in patients with long COVID. Some specific symptoms including dyspnea, palpitations, taste disorders, and olfactory disorders were associated with elevated BNP levels. We propose two possible reasons for BNP elevation in patients with long COVID: cardiac dysfunction and inflammation-mediated etiology. To further characterize BNP as a long COVID biomarker, additional studies, including evaluations of cardiac profiles and changes in BNP levels over time, are needed.

## Figures and Tables

**Table 1:** Clinical characteristics of 428 long COVID patients with known plasma BNP levels.

Patients’ Groups	Normal BNP (n = 314, 73.4%)	Increased BNP (n = 114, 26.6%)	*p*-Value
Patients’ profile			
Sex: male/female (%female)	175/139 (44.3%)	30/84 (73.7%)	<0.01 ** ^(a)^
Age (years), median [IQR]	38 [24–50.8]	51 [42.3–62]	<0.01 ** ^(c)^
BMI: (kg/m^2^), median [IQR]	22.1 [19.9–25.9]	22 [19.5–24.6]	0.45 ^(c)^
SBP: median [IQR]	121 [112–134]	128 [113–143]	<0.05 * ^(c)^
DBP: median [IQR]	73 [66.8–83]	73 [65.8–83]	0.09 ^(c)^
PR: median [IQR]	82 [74.5–91]	81 [74–88]	0.10 ^(c)^
Patient’s lifestyle			
Smoking habit, *n* (%)	74 (23.6%)	33 (28.9%)	0.25 ^(a)^
Alcohol drinking, *n* (%)	61 (19.4%)	30 (26.3%)	0.11 ^(a)^
Clinical management in acute phase of COVID-19			
Hospital admission, *n* (%)	13 (4.14%)	11 (9.65%)	<0.05 * ^(a)^
Oxygen therapy, *n* (%)	1 (0.32%)	3 (2.63%)	0.06 ^(b)^
Steroid therapy, *n* (%)	1 (0.32%)	2 (1.75%)	0.19 ^(a)^
Mild condition, *n* (%)	308 (98.1%)	106 (93%)	0.97 ^(b)^
Moderate to severe condition, *n* (%)	4 (1.27%)	7 (6.14%)	
COVID-19 vaccination status			
<2 doses, *n* (%)	75 (23.9%)	28 (24.6%)	0.67 ^(a)^
≥2 doses, *n* (%)	236 (75.2%)	82 (71.9%)	
Duration from the onset to the first visit			
Days: median [IQR]	108 [73.3–202]	135 [78–207]	0.16 ^(c)^

Medians [IQR: interquartile range] and percentages (%) are shown. BMI: body mass index, BNP: brain natriuretic peptide, COVID-19: coronavirus disease 2019, DBP: diastolic blood pressure, PR: pulse rate, SBP: systolic blood pressure. Normal BNP ≤ 18.4 pg/mL and high BNP > 18.4 pg/mL. (a) Pearson’s chi-square test, (b) Fisher’s exact test, or (c) a Mann–Whitney U test was performed. Differences were considered statistically significant at * *p* < 0.05 and ** *p* < 0.01.

**Table 2:** Patients’ backgrounds associated with increased BNP levels in long COVID patients.

	Odds Ratio	95% CI	*p*-Value
Age	1.06	1.04–1.07	<0.01 **
Female	3.80	2.22–6.50	<0.01 **
BMI (kg/m^2^)	0.98	0.94–1.04	0.61
Severity	1.65	0.80–3.40	0.18
Vaccination	0.72	0.40–1.28	0.2

BNP: brain natriuretic peptide, CI: confidence interval, COVID: coronavirus disease. A logistic regression analysis was performed. Differences were considered statistically significant at ** *p* < 0.01.

**Table 3:** Laboratory data for the normal BNP group and increased BNP group.

Patients’ Groups	Normal BNP (n = 314)	Increased BNP (n = 114)	Sample Defects	*p*-Value
Hb (g/dL)	14.7 (1.43)	13.5 (1.48)	(0; 0)	<0.01 **
Alb (g/dL)	4.52 (0.34)	4.24 (0.39)	(−5; 0)	<0.01 **
AST (U/L)	23.4 (24.1)	20.1 (7.23)	(−1; 0)	0.2
ALT (U/L)	27.4 (26.2)	21 (13.9)	(−1; 0)	0.17
ALP (U/L)	82.2 (44.8)	71.7 (20.7)	(−2; 0)	0.08
eGFR (mL/min/1.73 m^2^)	83.7 (17.4)	81.1 (20.3)	(−50: −3)	0.57
CRP (mg/dL)	0.13 (0.42)	0.21 (0.72)	(0; 0)	0.07
ESR (mm/h)	8.57 (8.04)	14.93 (20.84)	(−6; −1)	<0.01 **

Data are shown as mean (standard deviation: SD) values. Hb: hemoglobin, Alb: albumin, AST: aspartate aminotransferase, ALT: alanine aminotransferase, ALP: alkaline phosphatase, eGFR: estimated glomerular filtration rate, CRP: C-reactive protein, ESR: erythrocyte sedimentation rate. The Mann–Whitney U test was performed. Differences were considered statistically significant at ** *p* < 0.01.

## Data Availability

Detailed data will be available if requested from the corresponding author.

## Abbreviations

Albumin (Alb); aspartate aminotransferase (AST); alanine aminotransferase (ALT); alkaline phosphatase (ALP); body mass index (BMI); brain natriuretic peptide (BNP); chemiluminescent enzyme immunoassay (CLEIA); confidence intervals (CIs); coronavirus disease 2019 (COVID-19); COVID-19 aftercare clinic (CAC); C-reactive protein (CRP); erythrocyte sedimentation rate (ESR); estimated glomerular filtration rate (eGFR); hemoglobin (Hb); left ventricular ejection fraction (LVEF); multisystem inflammatory syndrome in children (MIS-C); myalgic encephalomyelitis/chronic fatigue syndrome (ME/CFS); N-terminal pro-B-type natriuretic peptide (NT-proBNP); odd ratios (ORs); and orthostatic intolerance (OI).
